# Effect of Different Cooling Regimes on the Mechanical Properties of Cementitious Composites Subjected to High Temperatures

**DOI:** 10.1155/2014/289213

**Published:** 2014-04-09

**Authors:** Jiangtao Yu, Wenfang Weng, Kequan Yu

**Affiliations:** College of Civil Engineering, Tongji University, Shanghai 200092, China

## Abstract

The influence of different cooling regimes (quenching in water and cooling in air) on the residual mechanical properties of engineered cementitious composite (ECC) subjected to high temperature up to 800°C was discussed in this paper. The ECC specimens are exposed to 100, 200, 400, 600, and 800°C with the unheated specimens for reference. Different cooling regimens had a significant influence on the mechanical properties of postfire ECC specimens. The microstructural characterization was examined before and after exposure to fire deterioration by using scanning electron microscopy (SEM). Results from the microtest well explained the mechanical properties variation of postfire specimens.

## 1. Introduction

Concrete is the most widely used construction material in the world. Although it was primarily designed for carrying compressive loads, concrete in real field conditions is also subjected to tensile stresses because of structural loading, shrinkage (if the shrinkage is restrained), chemical attack, and thermal deformations. The tensile strength of concrete is only approximately 10% of its compressive strength and brittle concrete cracks when subjected to tensile stresses. In recent years, the effort to modify the brittle nature of ordinary concrete has resulted in modern concepts of ultrahigh performance fiber-reinforced cementitious composites (UHP-FRCC), which are characterized by tensile strain-hardening after first cracking. Depending on its composition, its tensile strain capacity can be up to several hundred times of those normal and fiber-reinforced concrete. The engineered cementitious composite (ECC) is a special type of UHP-FRCC designed based on micromechanical principles to strain-harden in tension. It offers high ductility under uniaxial tensile loading and improved durability due to an intrinsically tight crack width of less than 100 *μ*m [1, 2]. During the last decade, the use of ECC has considerably grown up, and it was used in a variety of structures in various regions [3, 4].

It is well accepted that, in ECC mixture, partial Portland cement would be replaced by pozzolanic materials, particularly fly ash. For normal concrete, the partial replacement of Portland cement with pozzolanic materials, generally from 5% to 30% by mass, has been shown to improve the durability properties of blended cements [5–7]. This is largely attributed to the pozzolanic reaction, where reactive siliceous and aluminous phases react with portlandite to form new C–S–H or C–AS–H type phases. Importantly, the replacement of Portland cement with pozzolans reduces the overall CO_2_ footprint of the material. Emissions of CO_2_ are an important consideration in cement production since, due to its sheer scale, the industry accounts for 5–8% of global anthropogenic emissions [8].

Significant attention has been brought to the study of its rheological, mechanical, and durability related properties of ECC. With an increase in the application of ECC, the risk of exposure to elevated temperatures increases as well. The behavior of ECC exposed to high temperature has to be evaluated in particular.

For normal cement based composite, high temperatures caused physical and chemical changes, resulting in its mechanical property deterioration, such as compressive strength and modulus of elasticity. The residual properties of ECC after exposure to high temperatures have been studied by several researchers, mainly on the heating temperatures, mineral admixtures of fly ash, and fiber influence [9, 10]. But there are still some aspects that remained to be studied. For normal cement composite, different cooling regimes, that is, cooling in air, quenching in water, or water spraying, had a significant influence on the residual mechanical properties of postfire composite [11, 12]. The main objective of this research is to gain a better understanding of the influence of cooling regimes on the postfire ECC specimens subjected to high temperature up to 800°C.

## 2. Experimental Studies

### 2.1. Materials, Mix Proportions, and Basic Mechanical Properties

The materials used in the production of ECC mixture were Type I Portland cement (C), class F fly ash (FA), sand, water, polyvinyl alcohol (PVA) fibers, a polycarboxylic ether type high-range water-reducing admixture (HRWR), and Hydroxypropylmethyl cellose (HPMC). The mix details are given in Table 1. Unlike typical fiber-reinforced cementitious composites, the component characteristics and proportions within the ECC are carefully determined with the use of micromechanical design tools to achieve the desired strain-hardening response [13]. In this ECC mixture, about 30% of Portland cement was replaced by fly ash, which made it a kind of eco-friendly cementitious materials.

The PVA fibers with a diameter of 39 *μ*m and a length of 8 mm are purposely manufactured with a tensile strength (1620 MPa), elastic modulus (42.8 GPa), and maximum elongation (6.0%) matching those needed for strain-hardening performance. Additionally, the surface of the PVA fibers is coated with a proprietary oiling agent 1.2% by mass to tailor the interfacial properties between fiber and matrix for strain-hardening performance [13].

**Figure 1:**
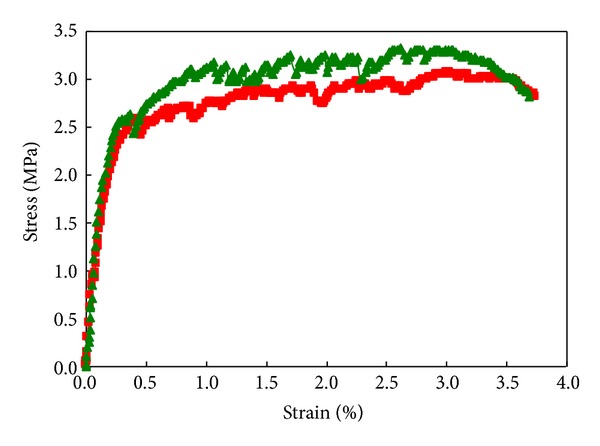
Typical tensile stress-strain response of ECC at 28 days.

To characterize the direct tensile behavior of the ECC mixtures, the dog-bone specimens were used. Direct tensile tests were conducted under displacement control at a loading rate of 0.005 mm/s. The typical tensile stress-strain curves of the ECC mixtures at 28 days are shown in Figure 1. As shown in Table 1, the ECC composites exhibited a strain capacity of 3.5% at 28 days, with an ultimate strength of about 3.3 MPa.

### 2.2. Test Specimen Preparation and Testing Procedure

Specimens were removed from the molds at 1 day and kept in a water tank until the age of 28 days. Five specimens were tested under compression immediately after conditioning; these control specimens will be referred to as those tested after exposure to normal curing condition (unheated).

**Figure 2:**
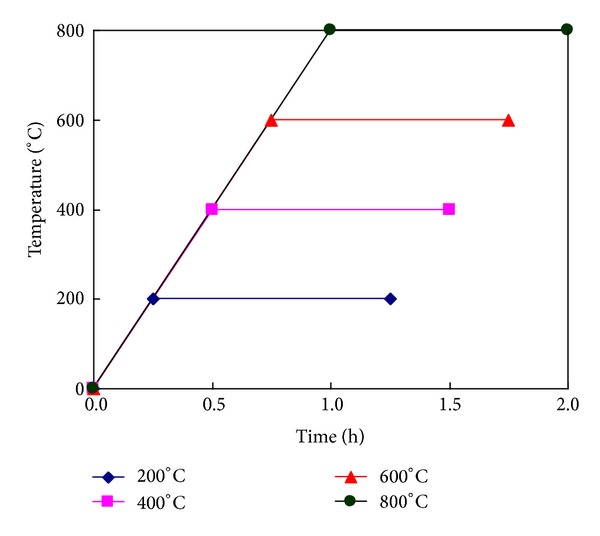
Heating regime for ECC specimens.

Computer controlled furnace was used for the heating of specimens with constant heating rate of about 13.3°C/min to reach the prescribed 100, 200, 400, 600, and 800°C temperature levels. The temperature was measured in the air at a position above the specimen inside the furnace. The temperature was maintained constant for an hour to achieve the thermal steady state condition after the target temperature is reached. It was expected that because of small size of specimens used in this study, the temperature in the center of the specimen reached the target temperature during the one-hour stabilization phase. The heating regime is shown in Figure 2. After heating, the samples were subjected to two cooling regimes as given in Table 2. After cooling, the specimens were sealed for 7 days before the compressive test. The test was performed under displacement control at a loading rate of 0.005 mm/s on a closed-loop controlled material testing system with 200 kN capacity. During the compressive tests, the load and the deflection values (obtained from a pair of LVDT's attached to the test set-up) were recorded on a computerized data acquisition system. Four samples were tested for each heating temperature and cooling regime. The weight of each specimen was also measured before and after exposure to calculate the mass loss of fire-deteriorated specimens.

The specimens are named as follows: temperature-cooling regime. For example, 400*R* (*W*), here *R* means cooling in air and *W* means cooling in water for 5 minutes.

## 3. Experimental Results and Discussions

### 3.1. Surface and Internal Characteristics

**Figure 3:**
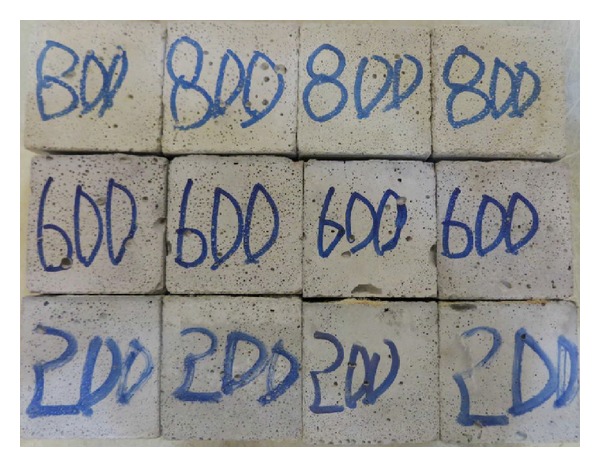
Color changes and surface cracks of postfire specimens.

It is observed that when the ECC specimens were exposed to high temperatures, some changes in color occurred.  Figure 3 shows that the color of ECC specimens changed from gray at 20°C to buff at 800°C due to the loss of water and chemical decomposition. The color of the specimens subjected to 800°C and then quenching in water turned to dark gray which may be due to further hydration of the composite.

Surface crack patterns of ECC specimens due to the high temperature exposure were almost same up to 800°C. Cracks became apparent after 400°C, and hairline cracks were monitored above 400°C. Increasing the exposure temperature to 800°C increased the size of microcracking as being consistent with pore structure variation of the specimens [9, 10]. However, quenching in the water helped to heal the surface crack due to further hydration.

### 3.2. Mass Loss and Water Absorption

**Figure 4:**
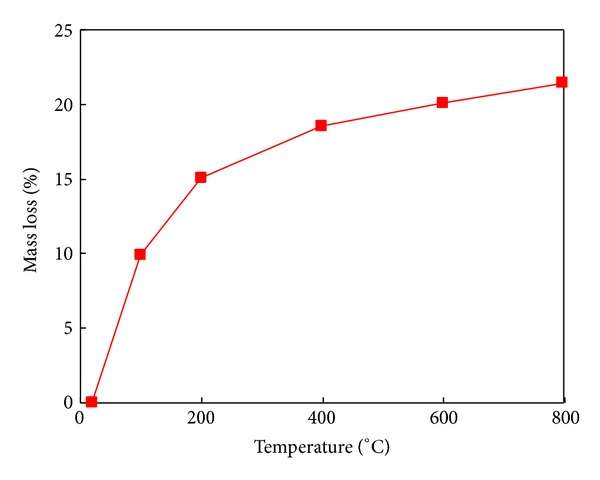
Mass loss of specimens of all curing ages with temperatures.

The deterioration of specimens subjected to various elevated temperatures was also assessed by mass loss measurements.  Figure 4 shows the relation between mass loss (*M*_*i*_/*M*_0_) (in percentage) and temperature of heat-exposed ECC. *M*_*i*_ is the mass after specific thermal heat exposure, and *M*_0_ is initial mass, prior to heat exposure. As seen in Figure 4, the mass loss increased with the increasing temperature of thermal exposure, a result mainly associated with the liberation of free and physically bound water. At higher temperatures of 600 and 800°C, the weight change of ECC was caused by the dehydration of paste [14]. During a heat treatment up to 400°C, the weight of the melted fibers also had an influence on mass loss.

For the specimens quenching in water, the water absorption was determined by weighting the mass variation before and after quenching. The water absorption rates were 9.96% and 24.14% for the specimens of 400*W* and 800*W*, respectively.

### 3.3. Microstructure Characterization by Using SEM Observations

**Figure 5:**
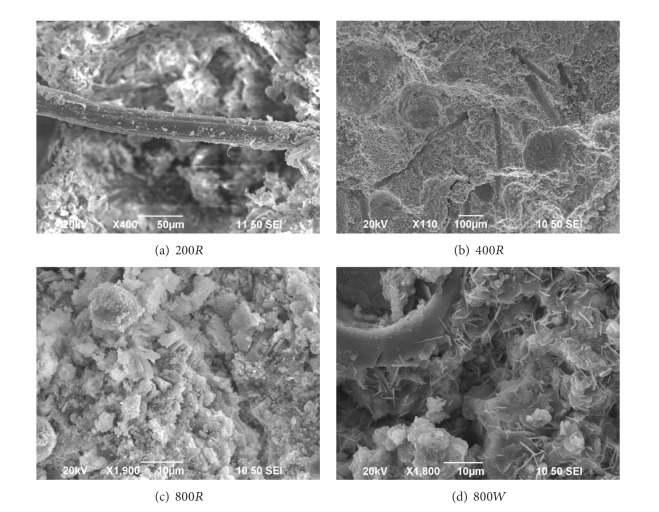
SEM micrograph of ECC specimens subjected to different heating temperatures and cooling regimes: (a) 200*R*; (b) 400*R*; (c) 800*R*; (d) 800*W*.

To study the behavior of fibers and matrix microstructure after various elevated temperatures, observations with an SEM were performed on samples taken from the core of postfire ECC specimens that had been exposed to temperature between 200 and 800°C for one hour. The specimens that had been quenched in the water for 5 minutes were also observed.  Figure 5 shows the SEM micrographs of various postfire ECC specimens exposed to different heating temperatures and cooling regimens. Figures 5(a) and 5(b) show the SEM micrographs of ECC specimens subjected to 200°C and 400°C cooling in air. The fiber did not melt when the specimens are subjected to 200°C. After exposure to 400°C, PVA fibers melt completely, creating additional interconnected pores and small channels in the matrix that fibers alone constitute a connected network. Therefore, the use of PVA fiber clearly affects porosity at high temperatures. After exposure to 400°C, the number and width of microcracks increase obviously compared to specimens subjected to 200°C from the SEM observation. Figures 5(c) and 5(d) show the micrographs of ECC specimens for 800*R* and 800*W*. After exposure to 800°C, the morphology of hydration products shows numerous microcracks and massive structure of hydration products; the hydration products are appearing as ill crystallized or amorphous structures by losing the characteristic crystal structure. However, Figure 5(d) shows the typical crystal structure of specimens quenching in water and curing for 7 days. The produce of new crystal enhances the strength of specimens which would be discussed in following section. Additionally, from the SEM observation, it is found that there is more new generated crystal in 800*W* specimens than in 400*W* specimens, which results in more significant mechanical increases in 800*W* specimens.

### 3.4. Residual Compressive Strength and Stress-Strain Curves

**Figure 6:**
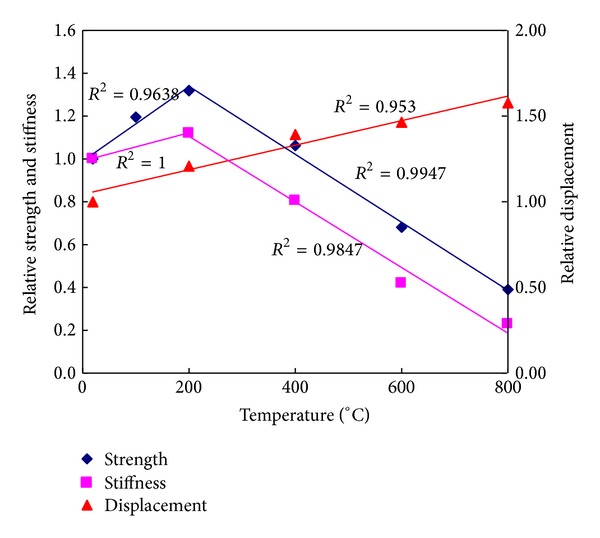
Compressive strength, stiffness, and displacement corresponding to the peak load with temperatures.

Figure 6 shows the influence of temperature on the compressive strength and stiffness of postfire ECC specimens. Each point in Figure 6 was obtained from the average of at least four test specimens. The coefficient of variance (COV) values for the compressive strength values ranged from 2.7% to 10.4%. The narrow range of COV values is an indication of the consistent repeatability of the compressive strength test method even for fire-deteriorated specimens.

As expected, exposure to high temperatures influenced the residual compressive strength of ECC specimens substantially. Percent variation in compressive strength can be classified in two distinct patterns of strength loss, 23–200°C and 200–800°C. Temperatures no more than 200°C seem to help the strength increase. Mean compressive strength of 28-day ECC specimens increase by 32% after exposure to 200°C. It can be partially due to the strengthened cement paste during the evaporation of free water, which leads to greater Van der Waal's forces as a result of the cement gel layers moving closer to each other [15, 16]. Further hydration of cementitious materials is another important cause of the hardening of cement paste. Especially for FA composite, unhydrated PFA particles can reacted with calcium hydroxide and gels produced C–S–H like [14, 16]. Although high temperature induced more microcracks, the enhancement effect mentioned above totally overcame the damage caused by microcracks.

Beyond 200°C, the compressive strength decreases monotonously. However, the influence of high temperature exposure on the residual compressive strength is not prominent up to 400°C with the mean compressive strength still being increased by 6% after exposure to 400°C with the same reasons as mentioned before. This might be due to the less sensitivity of compressive strength to minor microcracks. Heating up to 400°C generated a relatively small amount of cracking, which did not cause any immediate loss of carrying capacity in compression because the slightly cracked concrete could work as a highly redundant structure [17]. Beyond 400°C, however, compressive strength dropped monotonously by 32% and 61% at 600°C and 800°C, respectively. According to the variation of the residual compressive strength, temperature of 600°C and above might be regarded as critical temperature range for the strength loss of ECC. When the temperature was raised to 600°C, decomposition of the major hydrate, known as tobermorite (gel), was inevitable [18], causing severe increase in the microstructure of its matrix and the loss of binder property. By comparing the data given in this paper with published work on normal concrete or fiber-reinforced concrete [15, 19–21] that the standard ECC mixture (by retaining more than 35% of its original compressive strength and 22% of its original stifness capacity ater exposure to peak temperatures of 800°C for 1 hour) performs similarly to or better than fire-damaged plain concrete with steel and/or polypropylene fibers exposed to similar elevated temperatures.

The present test results are in line with the findings of previous studies [9, 10]. The compressive strength variation with temperatures could be expressed by the following equations:

(1) fc=0.0017Tm+0.9847, 20°C≤Tm≤200°C  R2=0.9638,fc=−0.0016Tm+1.6542, 200°C≤Tm≤800°C R2=0.9947.

From Figure 6, it can be seen that the stiffness of postfire specimens shares a similar but more sensitive tendency [19, 20] with exposure temperatures, which also could be classified in two stages. The mean compressive stiffness of ECC specimens increased by 12% after exposure to 200°C, while it decreased by 20%, 58%, and 73% after exposure to 400, 600, and 800°C. The compressive stiffness variation with temperatures could be expressed by the following equations:

(2) fc=0.0007Tm+0.9867, 20°C≤Tm≤200°C,     R2=1,fc=−0.0015Tm+1.3907, 200°C≤Tm≤800°C,  R2=0.9947.

Additionally, the displacement corresponding to the peak load increases with temperatures, which could be expressed by

(3) fc=0.0007Tm+1.04, 20°C≤Tm≤800°C, R2=0.953.

**Figure 7:**
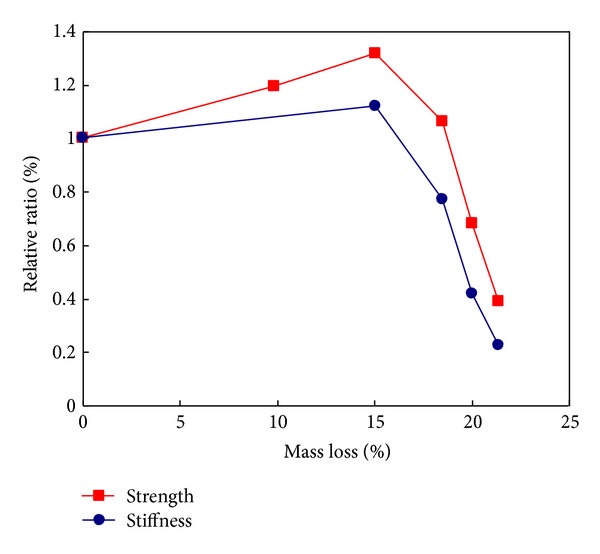
Relationships of strength and stiffness to mass loss.

Figure 7 shows the relationships between the mass loss and the mechanical properties, including the compressive strength and stiffness. It can be seen that the mechanical properties increased up to the mass loss around 15%, corresponding to the heating temperature of 200°C. Afterwards, the mechanical properties decreased with the increasing mass loss. The stifness is more sensitive than strength to mass loss or heating temperature.

**Figure 8:**
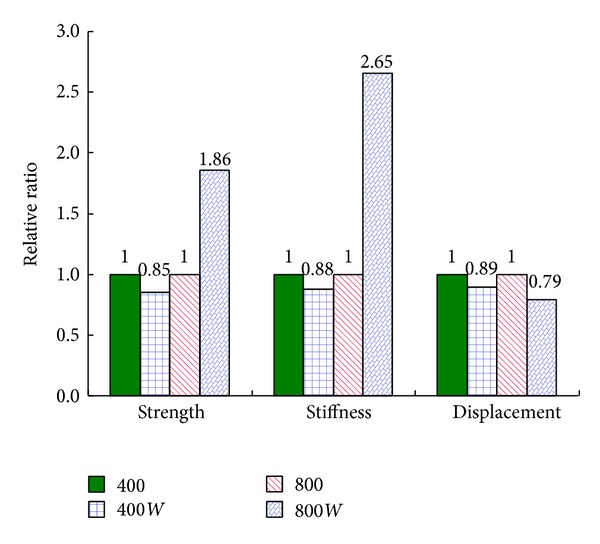
Influence of cooling regimes on compressive strength, stiffness, and displacement corresponding to peak load.

Figure 8 shows the influence of cooling regimes on the residual mechanical properties. For the specimens subjected to heating temperature of 400°C, the compressive strength and stiffness of specimens quenching in water decreased by 15% and 6% compared to the ones cooling in air, while, for 800°C, the compressive strength and stiffness of specimens quenching in water were 1.86 and 2.65 times to the ones cooling in air. As mentioned in Section 3.3, there is more new generated crystal in 800*W* specimens than in 400*W* specimens, which results in a more significant mechanical increase in 800*W* specimens. Meanwhile the displacement corresponding to the peak load of 400*W* and 800*W* deceased by 11% and 21% compared with the specimens cooling in air.

**Figure 9:**
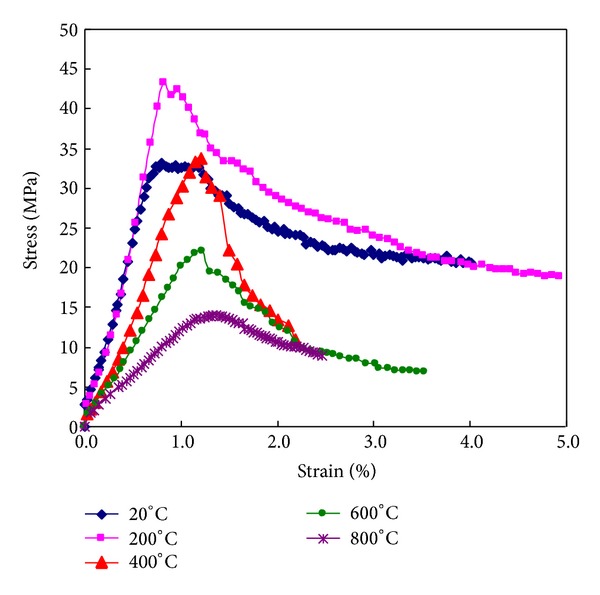
Post-fire specimens cooling in the air.

The stress-strain curves of ECC specimens at room temperature and elevated temperatures are compared in Figure 9. For temperature no more than 200°C, ultimate stress increased with the increasing temperature, particularly for heating temperatures of 200°C. For temperatures beyond 200°C, ultimate stress decreased with the increasing temperatures; however, the stress for 400°C was still a little higher than the one of unheated specimens. The slope decreased with the increase in exposure temperature up to 800°C, indicating a reduction in the stiffness of the ECC. The reduction in ECC stiffness was relatively low up to 400°C; however, beyond 400°C, a significant reduction was monitored in the ECC stiffness. As expected, with the increase of exposure temperature, the postpeak stress of the ECC specimens dropped faster, resulting in a smaller postpeak area under the curve. This behavior becomes more evident when the exposure temperature level reaches 800°C: ECC specimens failed soon after reaching their peak strength. This means that increasing the exposed temperature level tends to the ductile nature of ECC to brittle nature.

**Figure 10:**
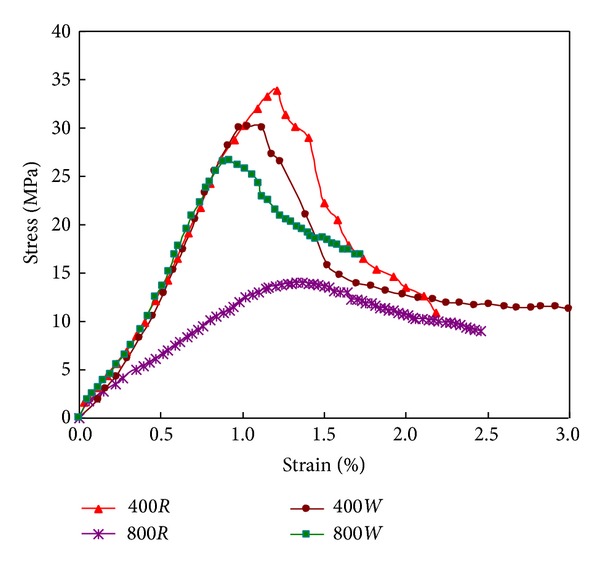
Stress-strain curve of specimens subjected to different cooling regimes.

Figure 10 shows the influence of cooling regimes on the stress-strain curves. The strength and stiffness of specimens subjected to 400°C and quenching in water for 5 minutes were slight lower than the ones cooling in air, while the strength and stiffness of quenching specimens were much higher than the ones of the specimens cooling in air for 800°C. As mentioned in Section 3.3, there is more new generated crystal in 800*W* specimens than in 400*W* specimens, which results in a more significant mechanical increase in 800*W* specimens.

## 4. Conclusions

The objective of the present work was to investigate the influence of cooling regimes on the mechanical properties of ECC specimens subjected to elevated temperatures. ECC specimens were exposed to heating temperature up to 800°C and subjected to two different cooling regimes, that is, cooling in air and quenching in water. The mechanical properties (compressive strength, stress-strain relationship, and stiffness) and microstructural properties (via SEM analyses) of ECC were studied at room temperature and postfire specimens. Based on this study, the following conclusions can be drawn.

1. The color of ECC specimens changed from gray at 20°C to light yellow at 800°C and the specimens quenching in water turned to dark gray. Hairline cracks were monitored above 400°C and quenching in the water may help to heal the surface crack due to further hydration.
2. The mass loss increased with the increasing temperature of thermal exposure, a result mainly associated with the liberation of free and physically bound water. At higher temperatures of 600 and 800°C, the weight change of ECC was caused by the dehydration of paste. During the heating treatment up to 400°C, the weight of the melted fibers also had an influence on mass loss.
3. The compressive strength can be classified in two stages of strength loss, 23–200°C and 200–800°C. Temperatures no more than 200°C seem to help the strength increase. Beyond 200°C, the compressive strength decreased monotonously with a drop of 61% at 800°C. The cooling regime of quenching in water helped the strength and stiffness recovery.
4. For temperature no more than 200°C, ultimate stress increased with the increasing temperature, while, beyond 200°C, ultimate stress decreased with the increasing temperatures. The slope decreases with the increase in exposure temperature up to 800°C, indicating a reduction in the stiffness of the ECC. Increasing the exposed temperature level tends to the ductile nature of ECC to brittle nature.

## Figures and Tables

**Table 1:** Mixture properties of ECC.

	ECC
Cement (C) (kg/m^3^)	650
Fly ash (FA) (kg/m^3^)	325
Water (W) (kg/m^3^)	375
PVA fiber (kg/m^3^)	26
Sand (kg/m^3^)	480
HRWR (kg/m^3^)	12
HPMC (kg/m^3^)	1.90
W/(C + FA)	0.38
FA/C	50%
28-day tensile strain (%)	3.5
28-day tensile strength (MPa)	3.3

**Table 2:** Two cooling regimes for different curing-age ECC specimens.

Temperature	Cooling at room temperature	Quenching in water
100	*✓*	None
200°C	*✓*	None
400°C	*✓*	*✓*
600°C	*✓*	None
800°C	*✓*	*✓*
